# Comparison of diagnostic accuracy of radiomics parameter maps and standard reconstruction for the detection of liver lesions in computed tomography

**DOI:** 10.3389/fonc.2024.1444115

**Published:** 2024-10-07

**Authors:** Alexander Hertel, Mustafa Kuru, Fabian Tollens, Hishan Tharmaseelan, Dominik Nörenberg, Nils Rathmann, Stefan O. Schoenberg, Matthias F. Froelich

**Affiliations:** Department of Radiology and Nuclear Medicine, University Medical Center Mannheim, Heidelberg University, Mannheim, Germany

**Keywords:** radiomics, radiomics maps, lesion detectability, liver metastases, colorectal cancer

## Abstract

**Background:**

The liver is a frequent location of metastatic disease in various malignant tumor entities. Computed tomography (CT) is the most frequently employed modality for initial diagnosis. However, liver metastases may only be delineated vaguely on CT. Calculating radiomics features in feature maps can unravel textures not visible to the human eye on a standard CT reconstruction (SCTR). This study aimed to investigate the comparative diagnostic accuracy of radiomics feature maps and SCTR for liver metastases.

**Materials and methods:**

Forty-seven patients with hepatic metastatic colorectal cancer were retrospectively enrolled. Whole-liver maps of original radiomics features were generated. A representative feature was selected for each feature class based on the visualization of example lesions from five patients. These maps and the conventional CT image data were viewed and evaluated by four readers in terms of liver parenchyma, number of lesions, visual contrast of lesions and diagnostic confidence. T-tests and chi²-tests were performed with a significance cut off of p<0.05 to compare the feature maps with SCRT, and the data were visualized as boxplots.

**Results:**

Regarding the number of lesions detected, SCTR showed superior performance compared to radiomics maps. However, the feature map for firstorder RootMeanSquared was ranked superior in terms of very high visual contrast in 57.4% of cases, compared to 41.0% in standard reconstructions (p < 0.001). All other radiomics maps ranked significantly lower in visual contrast when compared to SCTR. For diagnostic confidence, firstorder RootMeanSquared reached very high ratings in 47.9% of cases, compared to 62.8% for SCTR (p < 0.001). The conventional CT images showed superior results in all categories for the other features investigated.

**Conclusion:**

The application of firstorder RootMeanSquared feature maps may help visualize faintly demarcated liver lesions by increasing visual contrast. However, reading of SCTR remains necessary for diagnostic confidence.

## Highlights

- Selected radiomics feature maps may increase the visual contrast of liver metastases compared with standard CT reconstructions, potentially improving detectability.

## Introduction

With more than 19 million new cases per year and up to 10 million deaths, cancer is among the most pressing global health issues worldwide (1–3). In the course of cancer progression, metastases are an essential driver of cancer-related mortality, especially in the case of liver metastases (4). Therefore, early and valid detection of malignant liver lesions is favorable for sufficient therapy of patients.

Due to the metastatic spread through the portal venous system, gastrointestinal (GI) cancers, particularly colon cancer, are prone to developing liver metastases. Although the superior diagnostic accuracy of MRI with hepatocyte-specific contrast agents is known (5), computed tomography is still employed in the majority of patients, partly due to cost considerations (6). CT imaging can be problematic for detecting metastatic liver lesions due to suboptimal soft tissue contrast (7). Especially in the early development of metastases or after a response to therapy, liver lesions may appear faint despite the administration of a contrast agent, resulting in a lower diagnostic performance when compared to MRI and PET/CT (8). Yet, CT remains the commonly performed modality for the primary staging of metastases. Therefore, additional techniques and image analytics would be desirable to improve the sensitivity and the visual delineation of liver metastases.

Radiomics has emerged as a promising tool in medical imaging, offering the potential to extract quantitative features from standard imaging modalities that are not visible to the human eye. These features can provide insights into the underlying tissue characteristics, potentially leading to improved diagnostic accuracy. Several studies have demonstrated the advantages of radiomics in various clinical applications. Radiomics offers a promising approach in oncologic imaging by providing quantitative data that can reveal tumor characteristics not visible on conventional imaging techniques like standard CT reconstructions (SCTR). For example, radiomics has been shown to be effective in predicting treatment response and patient outcomes across various cancer types. In gastrointestinal cancers, radiomics has demonstrated its utility in diagnosing and staging tumors, predicting prognosis, and assessing response to therapy, particularly in cases where traditional imaging methods may fall short. Studies have shown that radiomics can reflect tumor heterogeneity, which is crucial for tailoring treatment strategies and improving patient management (9). Furthermore, radiomics has been used to assess the aggressiveness of liver tumors and predict patient survival, offering a non-invasive complement to traditional biopsy methods, which often have limitations such as sampling bias and procedural risks (10). These applications illustrate the potential of radiomics to enhance diagnostic precision and provide more personalized care, thereby overcoming some of the limitations associated with SCTR.

In this regard, the increasing importance of radiomics analysis of CT image datasets with the ability to generate visualized parameter maps of previously segmented lesions may provide new opportunities to facilitate the diagnosis of difficult-to-define lesions in the liver and potentially improve detectability (11–13). Therefore, the study aimed to investigate the diagnostic confidence and visual distinguishability for metastatic colorectal cancer (mCRC) liver metastases on radiomics feature maps compared to SCTR images.

## Materials and methods

### Study protocol

Forty-seven patients were retrospectively included in our data analysis based on the following inclusion criteria: the selected patients had known colorectal liver metastases confirmed in histopathology. Corresponding CT images were acquired as part of the clinical routine using iodine contrast agent (Imeron^®^ 350, Bracco IMAGING Deutschland GmbH, Konstanz, Germany) on commercially available clinical CT scanners (SOMATOM Emotion, SOMATOM Go Up ^®^, SOMATOM Flash, Siemens Healthcare GmbH, Erlangen, Germany). Patient data are presented in **Table 1**. The Institutional Review Board (2020-861R) of the Ethik Kommission II at the University Medical Center Mannheim, Germany, approved the proposed retrospective study protocol and the study was conducted in accordance with the Declaration of Helsinki. Image data were acquired according to local standard operating procedures with the following settings: Slice thickness of 1.5 mm, axial reconstruction, 130 -kV tube voltage, tube current modulation enabled, B30s kernel, pitch of 1.0, FOV of 400 mm, matrix 512×512, and pixel size of 0.78 mm × 0.78 mm.

**Table 1:** Patient characteristics.

Variable		Overall
*n*		47
Age at CT (median [IQR])		65.79 [56.99, 74.62]
Sex (%)		
	F	17 (36.2%)
	M	30 (63.8%)
T-Stage (%)		
	T1	2 (4.3%)
	T2	4 (8.5%)
	T3	24 (51.1%)
	T4	15 (31.9%)
	Tx	2 (4.3%)
N-Stage (%)		
	N0	8 (17%)
	N1	18 (38.3%)
	N2	20 (42.6%)
	Nx	1 (2.1%)
M-Stage (%)		
	M1	47 (100%)

### Image analysis workflow

Manual whole-liver segmentation was performed using MITK workbench (MITK Workbench v2021.02, Deutsches Krebsforschungszentrum, DKFZ, Heidelberg, Germany). The results were saved as a compressed nifti (nii.gz) data. Using a custom Docker container (Docker Desktop; Version 4.3.1, Docker, Inc., Palo Alto, CA, USA) based on pyradiomics (14) (version 3.0.1) scripts, the radiomics feature maps for the set of original radiomics features were calculated. The feature extraction process was conducted using the default settings in pyradiomics and adheres to the guidelines set by the Image Biomarker Standardization Initiative (IBSI) (15). The extraction involved gray-level discretization with a fixed bin width of 32 bins, resampling the images to a voxel size of 1 mm³, and applying a mask dilation with a radius of 1 voxel. The radiomic features extracted encompass various feature families, including first-order (FO) statistics, shape-based (SH) features, gray level co-occurrence matrix (GLCM), gray level run length matrix (GLRLM), gray level size zone matrix (GLSZM), neighboring gray tone difference matrix (NGTDM), and gray level dependence matrix (GLDM). KernelRadius was set to its default value of 1, and the voxelBatch parameter was set to “None”. MaskedKernel setting was set to “True”.

### Radiomics feature selection

**Figure 1:**
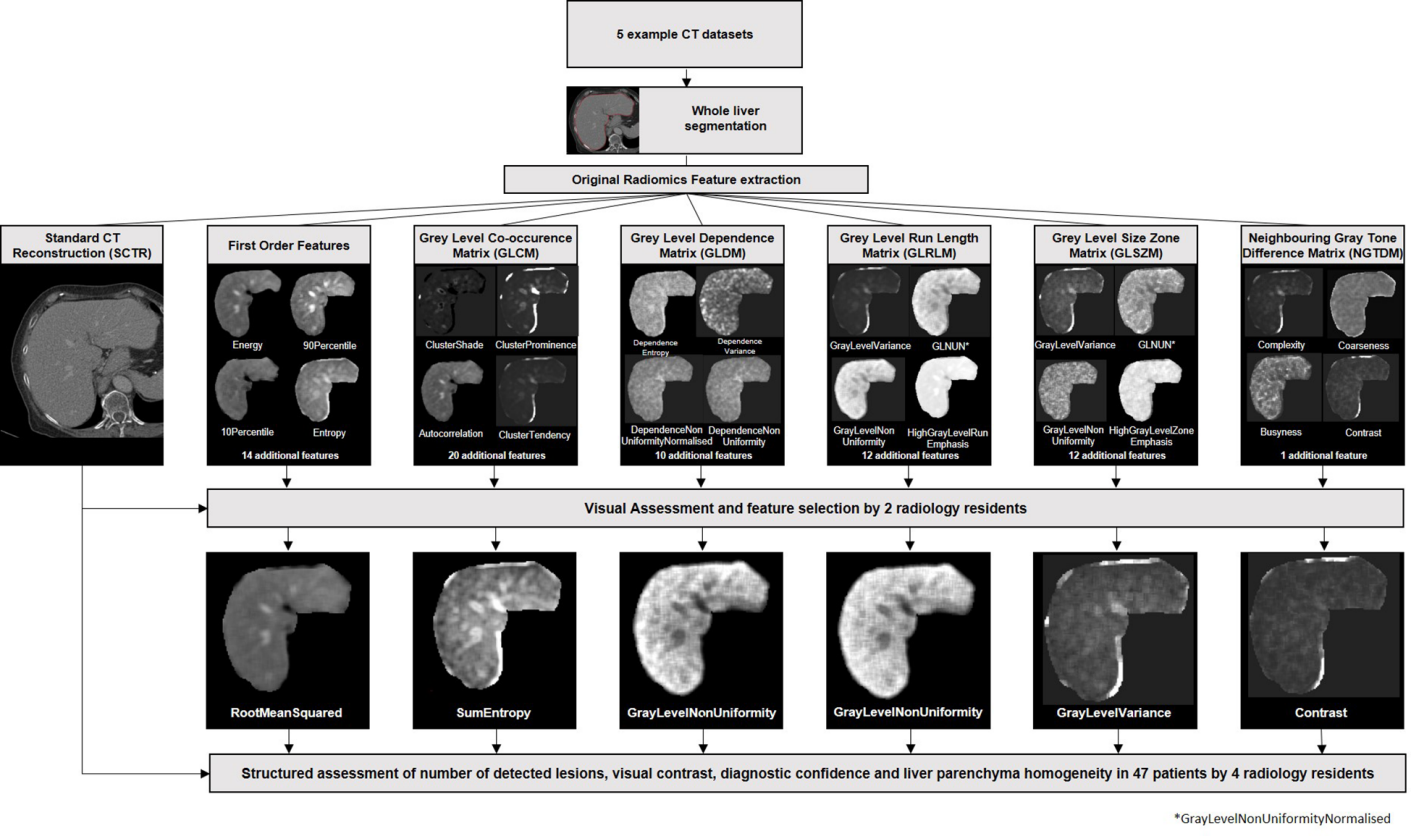
Study protocol. Whole liver segmentations were performed in five example CT datasets. Standard radiomics features were extracted and visualized parameter maps. Based on visual assessment by two readers, one feature of each feature class was selected. Subsequently, all parameter maps as well as the standard CT reconstructions of all patients were evaluated by four readers in a structured manner and evaluated according to the number of lesion, visual contrast, diagnostic confidence, and liver parenchyma homogeneity.

Feature maps for all original radiomics features were reviewed in five example patients by two radiology residents with 4 years and 2 years of experience in oncologic imaging and quantitative image analysis as preparation for the next step of feature selection: one representative parameter from each of the different classes (FO, GLCM, GLDM, GLRLM, GLSZM, and NGTDM) was selected based on different factors. The selection of radiomics features for this study was guided by their ability to enhance diagnostic accuracy in detecting and characterizing liver metastases. Features were prioritized based on their capacity to improve visual contrast between liver parenchyma, metastatic lesions, and anatomical landmarks, which is crucial for accurate lesion identification. Additionally, features demonstrating low variability in healthy liver tissue were selected to reduce the risk of false positives. One representative feature from each radiomics feature class was selected to ensure a comprehensive evaluation of the diverse types of textural information provided by different feature families. This approach ensured that the selected features were both practically effective and clinically relevant. A structured multi-reader analysis was performed for these six parameter maps and SCTR. To avoid bias, the readers examined the different radiomics maps in a predefined order that was staggered for each reader and rotated per patient. The study protocol and workflow are summarized in **Figure 1**. The reading criteria are shown in **Supplementary Table 1**.

### Parameter maps reading

A master table was created for the evaluation of the selected parameter maps. Homogeneity of liver parenchyma, number of metastases, contrast with surrounding parenchyma, and diagnostic confidence was evaluated based on a dedicated scheme (see the **Supplementary Material**). In addition, the contrast of the parameter maps was compared with that of conventional CT images and evaluated. To minimize the influence of window settings on lesion detectability, standardized window settings were used for each radiomics map, and radiologists did not alter these settings during the evaluation. All relevant image data (six radiomics feature maps plus SCTR per patient) of the 47 selected patients were assessed by four radiology residents with more than 2 years of experience in oncologic CT imaging.

### Statistical analysis

All data analyses were performed with R statistics in R Studio (version 4.1.0) (16). Plots were created with the package ggplot2 (17). Statistical analysis was performed on the pooled results from all radiologists. Each radiologist independently assessed the images, and their evaluations were then combined for the statistical analysis to provide an overall assessment of the performance of the radiomics feature maps versus the standard CT reconstructions. The differences in the reading results of the various radiomics maps and SCRT were tested for significance using Student’s t-test for paired distributions and the chi-squared test as well as Kruskal–Wallis H-test. A cutoff of p below 0.05 was applied for statistical significance. Distributions were visualized as boxplots.

## Results

### Collective characteristics of the patients and selection of parameters for mapping

The patients' collective characteristics have been published previously (18). Patient characteristics relevant to this analysis are summarized in **Table 1**.

Based on a structured rating of visual contrast for five patients by two radiology residents, the original radiomics features firstorder RootMeanSquared, GLCM sum entropy, GLDM non-uniformity, GLRLM non-uniformity, GLSZM variance, and NGTDM contrast were selected for further analysis (**Figure 1**).

### Liver parenchyma and detected number of lesions

**Figure 2:**
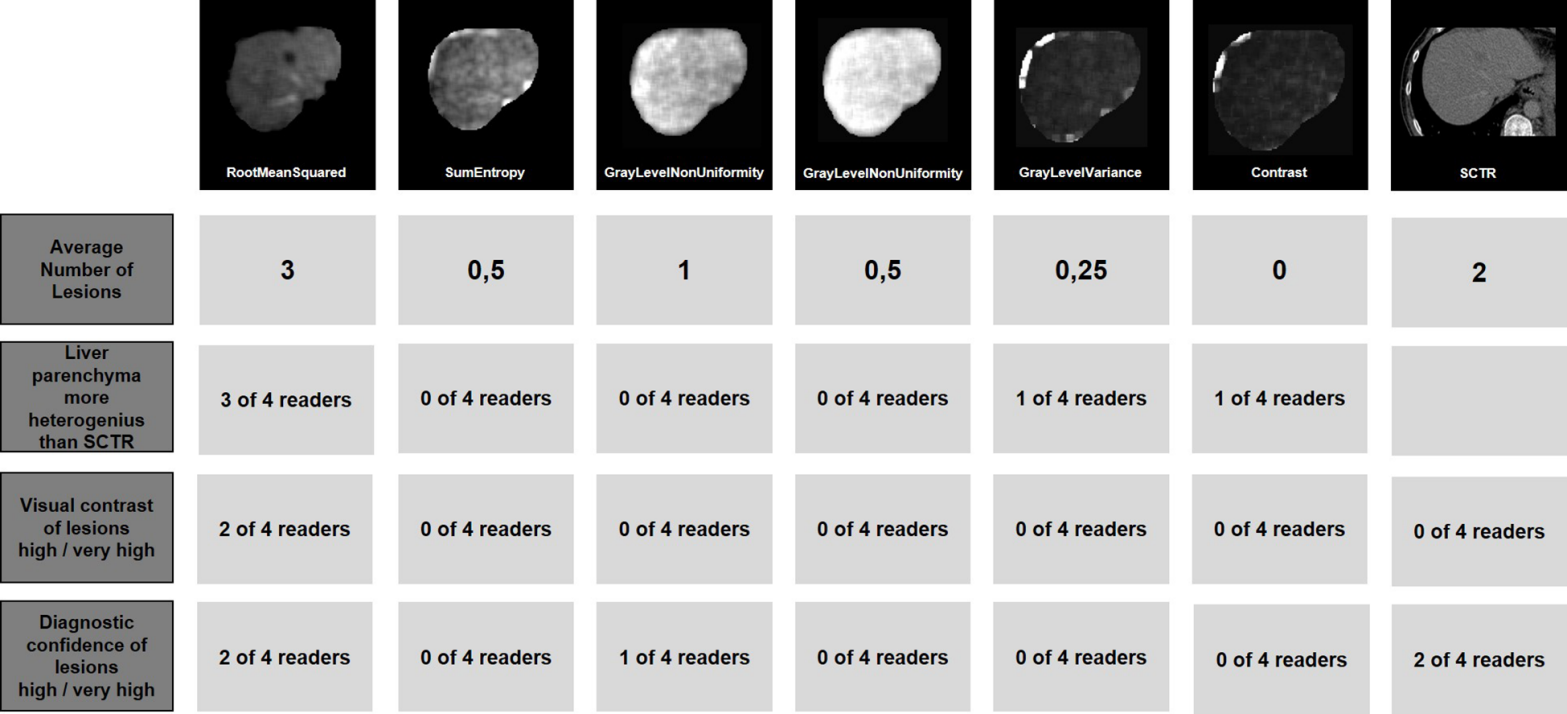
Example reading of one patient. The image section of an exemplary lesion is shown in the standard CT reconstruction as well as in all evaluated radiomics maps. In addition, the Average number of lesions is shown as well as the number of readers who rated the liver parenchyma as more heterogeneous than in the SCTR, the visual contrast as high or very high, and the diagnostic confidence as high or very high. Original firstorder RootMeanSquared shows a superior contrast for two liver lesions.

**Figure 3:**
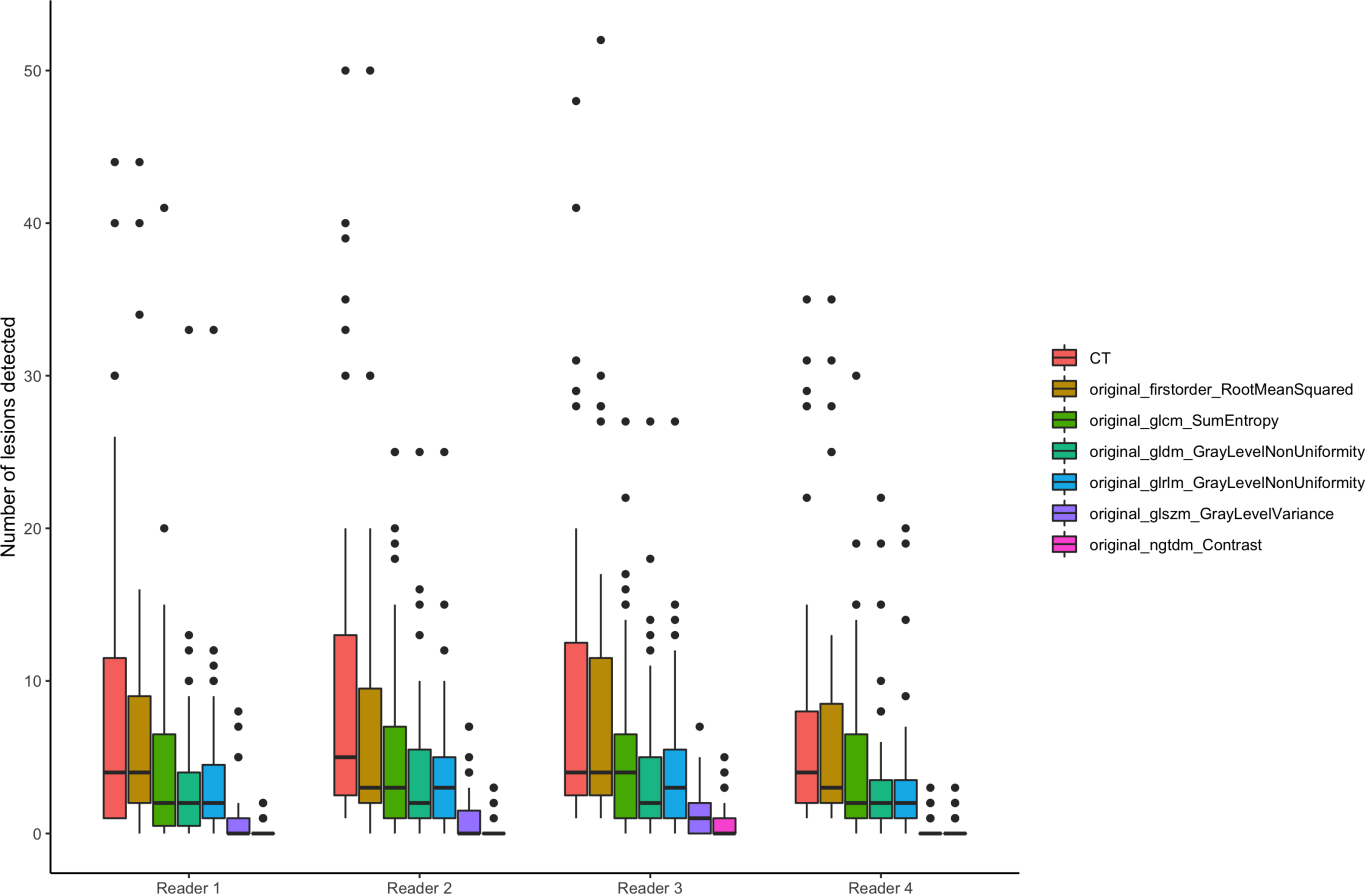
Number of detected lesions. Number of detected lesions by the four readers plotted by SCTR and radiomics features visualized as box plots.

**Figure 4:**
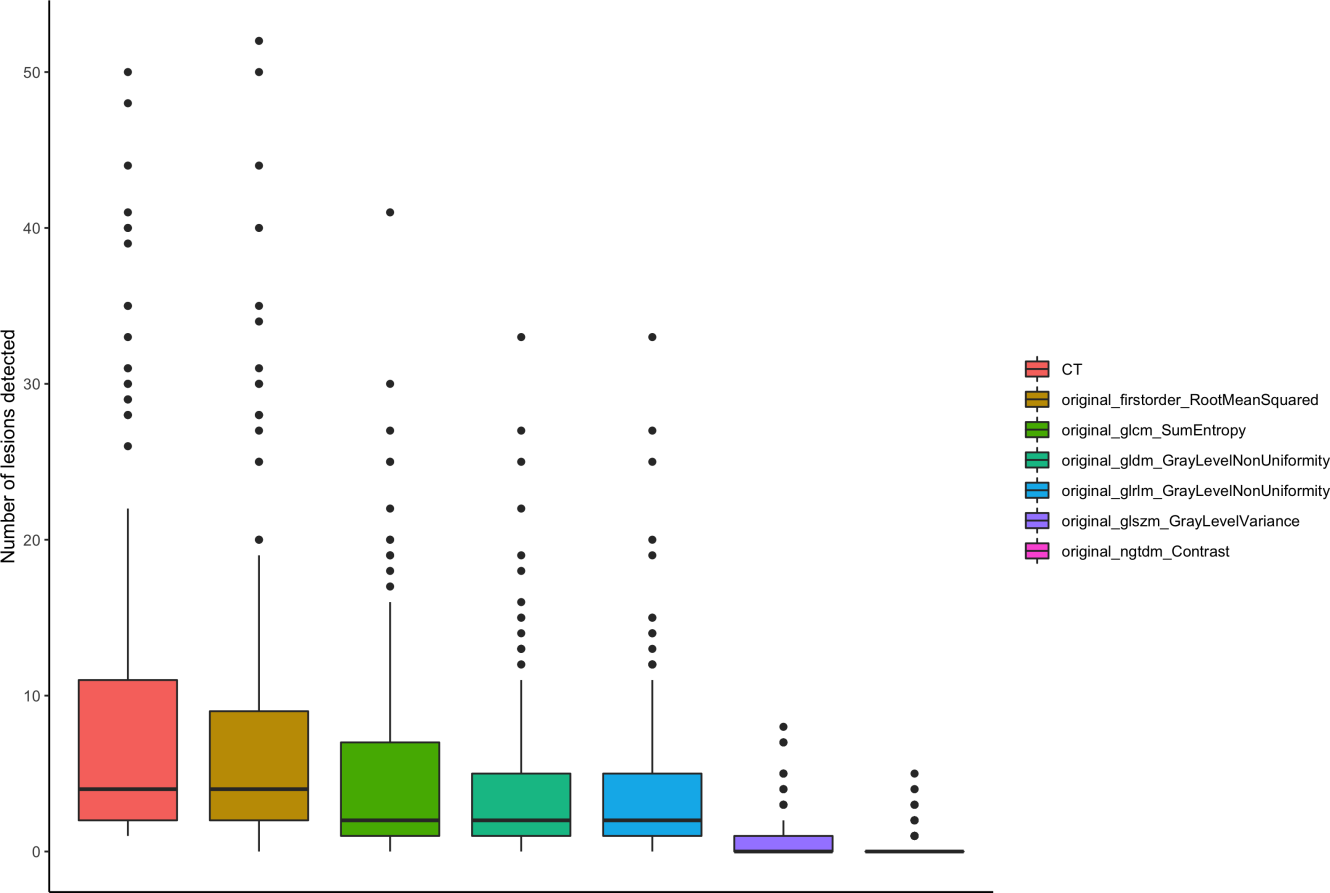
Number of lesions summarized for all readers. Number of lesions summarized for all readers plotted by SCTR and radiomics features visualized as box plots.

Overall, the liver parenchyma was more heterogeneous in all selected radiomics maps than conventional CT imaging except for firstorder RootMeanSquared (85.6% more homogenous, overall p < 0.001). Significantly fewer lesions were detected in the radiomics maps GLCM sum entropy, GLDM non-uniformity, GLRLM non-uniformity, GLSZM variance, and NGTDM contrast (e.g., 0.39 lesions on average in NGTDM contrast) compared to the conventional CT images (p < 0.001; **Figure 2**). Also, a lower detection rate was found in the firstorder RootMeanSquared maps, with an average number of 7.55 lesions detected per patient compared to 8.84 lesions detected in the conventional CT images (p < 0.001) (see **Table 2**). The number of lesions detected individually by the four readers is displayed in **Figures 3**, **4**. The p-value for the number of detected lesions between the individual features for each reader was <0.0001.

**Table 2:** Comparative performance of radiomics maps and standard CT imaging.

		(SCTR)reconstruction CTStandard	RootMeanSquared original_firstorder	sum entropy Original GLCM	non-uniformity Original GLDM	non-uniformity Original GLRLM	variance Original GLSZM	contrast Original NGTDM	p value
Total number of reads (4 readers × 47 patients)		188	188	188	188	188	188	188	
									
Parenchyma (%)	More heterogenous	NA	20 (10.6)	165 (87.8)	159 (84.6)	129 (68.6)	149 (79.3)	152 (80.9)	<0.001
	More homogenous	NA	161 (85.6)	20 (10.6)	24 (12.8)	52 (27.7)	38 (20.2)	34 (18.1)	
	Similar	NA	7 (3.7)	3 (1.6)	5 (2.7)	7 (3.7)	1 (0.5)	2 (1.1)	
Number of lesions [mean (SD)]		8.84 (10.69)	7.55 (9.52)	4.93 (6.50)	3.92 (5.18)	4.04 (5.02)	0.82 (1.56)	0.39 (0.89)	<0.001
Visual contrast (%)	0 (no lesions detectable)	0 (0.0)	7 (3.7)	33 (17.6)	34 (18.1)	30 (16.0)	123 (65.4)	147 (78.2)	<0.001
	1 (very low)	0 (0.0)	1 (0.5)	5 (2.7)	18 (9.6)	9 (4.8)	24 (12.8)	16 (8.5)	
	2 (low)	8 (4.3)	2 (1.1)	9 (4.8)	28 (14.9)	40 (21.3)	22 (11.7)	14 (7.4)	
	3 (medium)	36 (19.1)	20 (10.6)	62 (33.0)	51 (27.1)	58 (30.9)	15 (8.0)	5 (2.7)	
	4 (high)	67 (35.6)	50 (26.6)	64 (34.0)	52 (27.7)	43 (22.9)	2 (1.1)	3 (1.6)	
	5 (very high)	77 (41.0)	108 (57.4)	15 (8.0)	5 (2.7)	8 (4.3)	2 (1.1)	3 (1.6)	
Diagnostic confidence (%)	0 (no lesions detectable)	0 (0.0)	7 (3.7)	33 (17.6)	34 (18.1)	30 (16.0)	123 (65.4)	144 (76.6)	<0.001
	1 (very low)	0 (0.0)	2 (1.1)	7 (3.7)	23 (12.2)	17 (9.0)	29 (15.4)	23 (12.2)	
	2 (low)	9 (4.8)	8 (4.3)	18 (9.6)	33 (17.6)	38 (20.2)	23 (12.2)	12 (6.4)	
	3 (medium)	19 (10.1)	14 (7.4)	56 (29.8)	45 (23.9)	50 (26.6)	13 (6.9)	4 (2.1)	
	4 (high)	42 (22.3)	67 (35.6)	63 (33.5)	48 (25.5)	41 (21.8)	0 (0.0)	5 (2.7)	
	5 (very high)	118 (62.8)	90 (47.9)	11 (5.9)	5 (2.7)	12 (6.4)	0 (0.0)	0 (0.0)	

NA, not applicable.

### Visual contrast and diagnostic confidence

For visual contrast, firstorder RootMeanSquared achieved a very high ranking in 108 reads (57.4%) compared to 77 reads (41.0%) for SCTR (p < 0.001). However, for diagnostic confidence, firstorder RootMeanSquared achieved a very high ranking in 90 reads (47.9%) compared to 118 reads (62.8%) for SCTR (p < 0.001). All other radiomics features showed a significantly lower visual contrast and diagnostic confidence (**Table 2**).

## Discussion

This study compared radiomics feature maps to conventional CT reconstructions in terms of liver parenchyma visualization, liver lesion detection rate, visual contrast and diagnostic confidence. The conventional CT images performed significantly better than the selected radiomics maps concerning liver parenchyma homogeneity, lesions detected, and diagnostic confidence. However, firstorder RootMeanSquared showed a superior rating for visual contrast.

As the detectability of liver lesions strongly depends on the visual contrast of lesions in computed tomography, the increased contrast may help in lesions delineated faintly, especially in the case of inexperienced readers, by enhancing their sensitivity. In contrast, the comparative advantage of SCTR, in terms of diagnostic confidence, may be expected, as the standard CT reconstruction contains more visual information on the lesions’ properties for the reader. Since the radiomics maps differ significantly from the SCTR in terms of visual impression, this may be a possible explanation for the lower diagnostic certainty compared to the SCTR, with which radiologists have been familiar in routine practice for years. While some radiomics feature maps exhibited reduced resolution compared to SCTR images, which may have limited the detection of smaller liver lesions, others demonstrated an improved contrast between lesions and liver parenchyma, highlighting a trade-off between image resolution and contrast enhancement. Therefore, the application of firstorder RootMeanSquared feature maps would be recommended as an additional read, comparable to computer-aided diagnosis (CAD) systems, than as a replacement for SCTRs. The feature itself is defined as the square root of the mean of all squared intensity values. It is an indicator of the magnitude of the image values (19).

Radiomics feature maps have been applied for several use cases in oncologic imaging, especially in response assessment to medical treatment: A study by Correa et al. (20) investigated the association of glioblastoma lesion habitat with response to treatment. Several radiomics features could be identified that can be used to differentiate a low or high risk of treatment failure.

A similar approach has been successfully applied by Beig et al. (21) to the distinction between granuloma and adenocarcinoma: Using deep learning mechanisms of selected intra- and peri nodular radiomics features, differentiation between adenocarcinomas and granulomas is possible with an area under the curve (AUC) value of 0.8.

Penzias et al. (22) have shown that radiomics analysis of prostate MRI (T2w), especially by Gabor texture features, is feasible for predicting Gleason score in patients with prostate cancer with an AUC of 0.69.

In the work of Algohary et al. (23), T2w and apparent diffusion coefficient (ADC) sequences of prostate MRIs were radiomically evaluated, and selected radiomics features were presented as visualized maps. In the group of negative MRIs with positive biopsy (the MRIs had been assessed in accordance with the PI-RADS standard), carcinoma areas could be retrospectively identified in the radiomics maps, which could not be delineated in the mere T2w or ADC images.

However, the application of radiomics maps to improve the visual contrast of lesions has not been studied comprehensively. While this study demonstrates the potential of radiomics feature maps, particularly the firstorder RootMeanSquared map, to enhance the visual contrast of liver metastases on CT images, it also opens several avenues for future research. These findings could drive the development of more advanced radiomics-based diagnostic tools, which may complement SCTR. The application of such selected parameter maps could support radiologists in the routine interpretation of oncological imaging datasets, similar to how CAD systems assist in the detection of pulmonary nodules. Further research should focus on refining and validating these parameters across diverse clinical settings to ensure their robustness and reproducibility. Additionally, integrating these tools into clinical practice could lead to more personalized and accurate diagnostics, ultimately improving patient outcomes. Addressing these aspects will be crucial in realizing the full potential of radiomics in routine clinical use.

### Limitations

The implementation of radiomics feature maps in routine clinical practice presents significant challenges, both technical and organizational. The technical complexity of radiomics analysis requires specialized expertise in image processing and data analysis, necessitating extensive training for radiologists and technicians. Furthermore, integrating radiomics into existing clinical workflows poses an additional hurdle, as current systems and processes must be adapted to effectively incorporate these new technologies. Addressing these challenges requires interdisciplinary collaboration and continuous development of technology and training programs to facilitate the adoption of radiomics in clinical practice. Despite the promising potential of radiomics feature maps, their immediate applicability in everyday diagnostic settings is hindered by several factors. The lack of widespread validation and standardization of radiomics features poses a significant challenge, as it limits the reproducibility and generalizability of results across different clinical settings. Moreover, potential regulatory and cost barriers further complicate the integration of radiomics into routine practice.

Furthermore, while this study demonstrates the potential of radiomics maps, it also reveals the poor performance of certain radiomics features compared to standard CT reconstructions. This discrepancy may be attributed to several factors, including the inherent variability in feature extraction, the influence of image acquisition parameters, and the complexity of interpreting radiomics features in heterogeneous tissues such as liver parenchyma. Identifying and addressing these factors are crucial for enhancing the performance and clinical utility of radiomics. Future research should focus on optimizing feature selection and standardization processes, as well as improving the robustness of radiomics features to better match or exceed the diagnostic confidence provided by conventional imaging techniques.

Regarding the analysis of different radiomics maps, further limitations arise particularly in cases where the metastases are immediately subcapsular to the margin of the liver. These cannot be delineated partly for technical shortcomings in the segmentations and the basic calculation of the radiomics features due to the density differences between liver parenchyma and surrounding soft tissue. This problem could be addressed by methodologically optimized segmentation and radiomics map generation techniques. Since this study is a methodologically novel approach, optimized segmentation and visualization techniques must be further explored in future research. Additionally, the selection of representative features based on visual contrast in a subset of five patients may introduce bias, potentially limiting the generalizability of the findings.

## Conclusion

In summary, conventional CT images are superior to the selected radiomics feature maps GLCM sum entropy, GLDM non-uniformity, GLRLM non-uniformity, GLSZM variance, and NGTDM contrast in terms of homogeneity of liver parenchyma, detectability of liver lesions, visual contrast as well as diagnostic confidence. Only firstorder RootMeanSquared maps showed a comparable lesion detection rate. Furthermore, visual contrast was significantly more often rated as very high in the latter than in the SCTR. In the case of liver lesions that are only faintly delineated on conventional CT imaging, evaluating firstorder RootMeanSquared maps may offer an additive advantage in terms of detectability.

## Data Availability

The raw data supporting the conclusions of this article will be made available by the authors, without undue reservation.

## Supplementary material

Supplementary Figure 1 Exemplary high-resolution representation of several feature maps.

Supplementary Figure 2 High-resolution images of the liver maps of a liver lesion that can be faintly delineated in the SCTR.
