# Supplementary figures and images for: Comparison of diagnostic accuracy of radiomics parameter maps and standard reconstruction for the detection of liver lesions in computed tomography
Source: Front Oncol. 2024 Oct 7;14:1444115. doi: 10.3389/fonc.2024.1444115 (PMC11491382; doi:10.3389/fonc.2024.1444115)

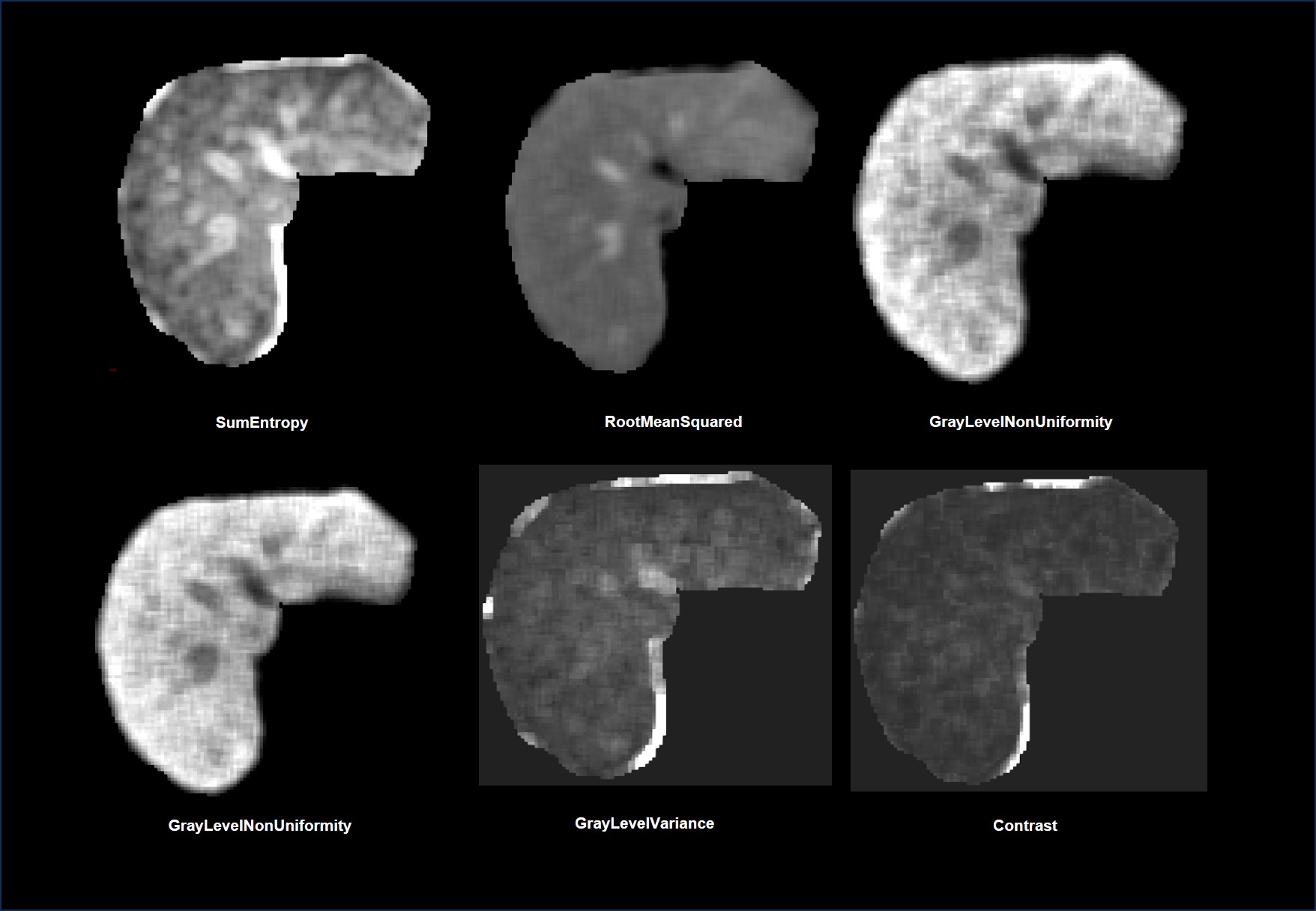

Supplement: Supplementary Figure 1 — Exemplary high-resolution representation of several feature maps. [file Image1.jpeg]

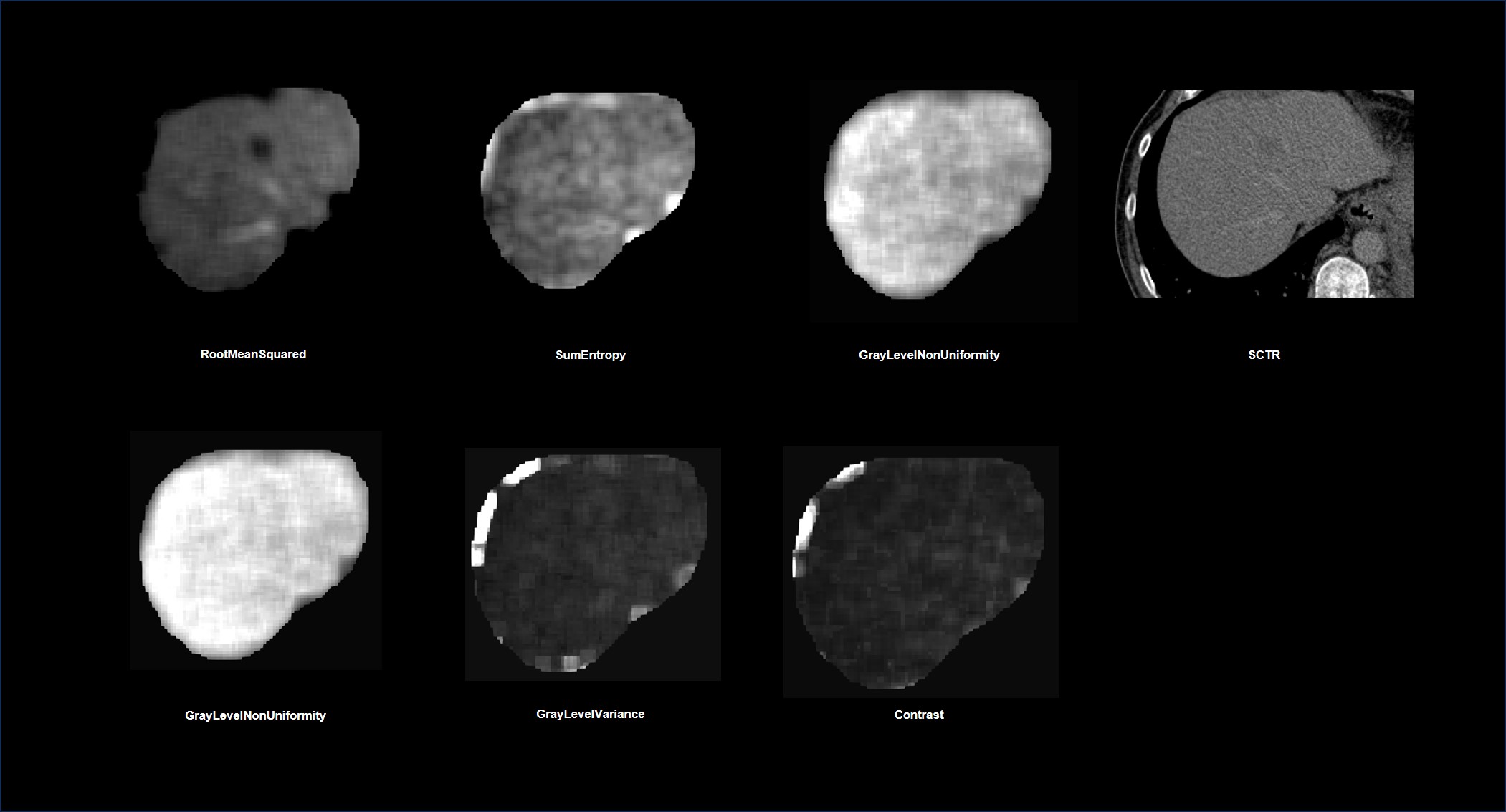

Supplement: Supplementary Figure 2 — High-resolution images of the liver maps of a liver lesion that can be faintly delineated in the SCTR. [file Image2.jpeg]
